# Current status for utilization of cold resistance genes and strategies in wheat breeding program

**DOI:** 10.3389/fgene.2024.1473717

**Published:** 2024-10-22

**Authors:** Shijie Ma, Xiaorong Huang, Xiaoqing Zhao, Lilong Liu, Li Zhang, Binjie Gan

**Affiliations:** Crop Research Institute, Anhui Academy of Agricultural Sciences, Hefei, Anhui Province, China

**Keywords:** *Triticum aestivum* L., cold resistance, gene, mechanism, breeding strategy

## Abstract

Low temperature chilling is one of the major abiotic stresses affecting growth and yield of *Triticum aestivum L*. With global climate change, the risk of cold damage in wheat production has increased. In recent years, with the extensive research on wheat chilling resistance, especially the development of genetic engineering technology, the research on wheat chilling resistance has made great progress. This paper describes the mechanism of wheat cold damage, including cell membrane injury, cytoplasmic concentration increased as well as the imbalance of the ROS system. Mechanisms of cold resistance in wheat are summarised, including hormone signalling, transcription factor regulation, and the role of protective enzymes of the ROS system in cold resistanc. Functions of cloned wheat cold resistance genes are summarised, which will provide a reference for researchers to further understand and make use of cold resistance related genes in wheat. The current cold resistant breeding of wheat relies on the agronomic traits and observable indicators, molecular methods are lacked. A strategy for wheat cold-resistant breeding based on QTLs and gene technologies is proposed, with a view to breeding more cold-resistant varieties of wheat with the deepening of the research.

## 1 Introduction

Wheat (*Triticum aestivum L.*) is one of the world’s top three grain crops. According to the FAO, around 200 million hectares of wheat will be planted globally with a production of about 787 million tonnes in 2023. Wheat is the calorie source for one-fifth of the global population (Pang et al., 2021), therefore, high and stable wheat production plays a crucial role in global food security. In recent decades, the likelihood of extreme weather on Earth has increased dramatically as greenhouse gases continue to be emitted (Jiang et al., 2022), low-temperature freezes occur from time to time, posing a significant threat to wheat production (Sutka, 2001; Vij and Tyagi, 2007). It occurs not only in the main temperate wheat-producing regions, but also in subtropical and Mediterranean climatic regions, covering the main wheat-producing countries such as China, the United States, Canada, Australia and some parts of South America. In the 25 years since 1990, the frequent occurrence of wheat freezes has caused severe losses over large areas in the main wheat-producing regions of central China, with the most severe reductions in yields of up to 30% (Yang and Yuan, 2014). In the central regions of the United States, statistics from 1955 to 2010 show that the yield of wheat is reduced by 210 kg per hectare due to low-temperature frosts (Holman et al., 2011). In the UK and eastern Australia, there has also been a marked increase in recorded frosts since the 1950s, causing some damage to wheat production (Whaley et al., 2004; Zheng et al., 2015). The incidence of frost damage results in millions of tonnes of food production being lost each year, with direct economic losses in excess of tens of billions of dollars. Wheat growth and development will be subjected to more cold stresses as the global climate continues to change, so improving varietal cold tolerance is very important in wheat breeding (Pang et al., 2021).

Wheat is a winter-habituated crop that must experience low temperatures to complete vernalisation (Fowler and Carles, 1979; Fowler et al., 1996), during inter-annual growth, they are exposed to the effects of multiple cold stress. The wheat is in the seedling stage before the winter, it will appear leaves dry, root damage or even dead seedlings phenomenon in case of a sudden drop in temperature and the yields will be also affected. After winter, if wheat is weakly resistant to cold the low-temperatures can damage plant tissues and inhibit growth and development (Subedi et al., 1998; Barton et al., 2014). During the period from nodulation to tasseling, wheat is in a period of parallel nutritive and reproductive growth, spikes can become hollow or underdeveloped as a result of frost or “spring cold” (Limin and Fowler, 2006), which will have a disastrous effect on wheat production. In summary, wheat is often exposed to low temperatures at critical stages of growth and development, and data shows that nearly 85% of the world’s wheat area is affected by spring cold each year (Ferrante et al., 2021), it also shows the practical importance of research on cold resistance in wheat for production. The research on the function of plant cold resistance genes has been widely carried out with the deepening of research on the mechanism of plant cold resistance and plant genetic engineering. In this review, we will summarise the achievements in the mining and utilisation of cold-resistant genes in wheat and clarify the physiological and molecular mechanisms of cold resistance, and strategies for wheat cold resistance breeding will be provided.

## 2 Research progress of cold resistance mechanism in wheat

**FIGURE 1:**
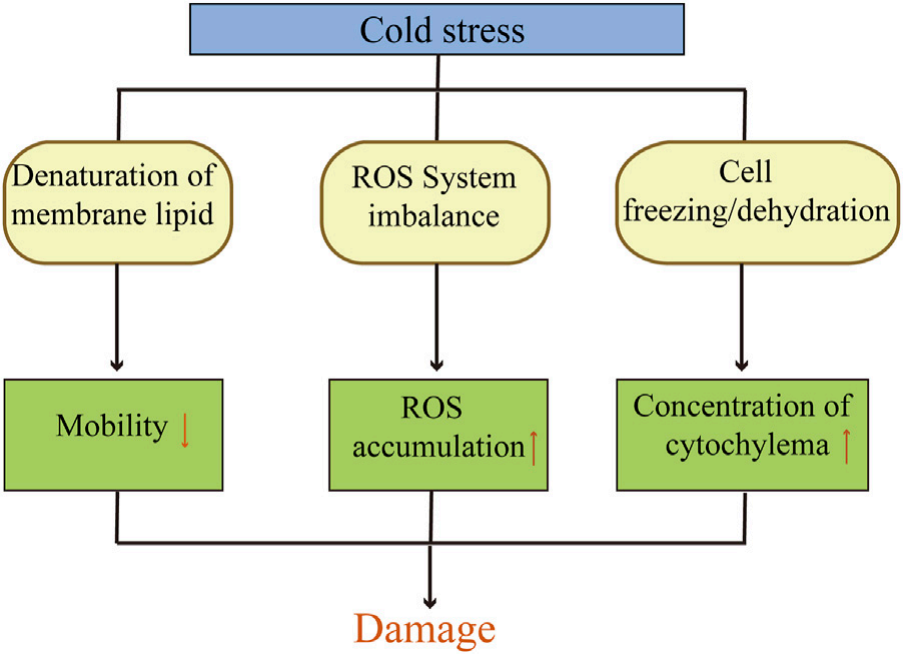
Mechanism of damages in wheat under cold stress. ↑ represent upward; ↓ represent down.

Low temperature affect the physiological and biochemical responses of plant cells seriously. Numerous studies have shown that the cold resistance of wheat is closely related to the mobility of cell membrane, reactive oxygen system and cellular osmoregulation. The mechanism of damages in wheat under cold stress is shown in Figure 1. The response to low-temperature involves multiple levels of regulation in wheat, including transcriptional regulation, hormonal regulation and signal transmission. These regulatory processes are quite complex and involve a large number of genes related to cold resistance.

### 2.1 Physiological mechanism of cold resistance in wheat

After suffering from cold damage, wheat cell tissues can be damaged or even die, mainly due to the destruction of cell membrane structures (Lyons, 1973). The stability of cell membranes is determined by the composition of membrane lipid fatty acids. The stability of cell membrane is determined by the fatty acid composition of membrane lipids. Generally, a higher content of unsaturated fatty acids in membrane lipids correlates with an enhanced plant cold resistance. Therefore, fatty acid desaturase (FAD)plays a certain role in plant cold resistance (Ishizaki-Nishizawa et al., 1996; Kodama et al., 1995). The cell membrane also serves as a sensor and conduit for low-temperature signals, encompassing calcium signals, protein kinases, phosphatases, and transcription factors, all of which participate in the cold stress signaling pathway (Soleimani et al., 2022). Researchers indicated that the electrical conductivity method for determining the permeability of cell membranes of winter wheat leaves under indoor cold stress showed a significant positive correlation with the cold resistance of wheat in the field (Ju et al., 2012).

The reactive oxygen species (ROS) accumulation and scavenging system in plants is crucial for maintaining the normal cellular functions. Generally, the accumulation and scavenging of ROS in this system is in dynamic balance. Persistent low temperatures can disrupt this balance, accelerating ROS accumulation and producing a large amount of free radicals, which in turn cause physiological disorders within cells (Zeng et al., 2011; Bhattacharjee, 2005). SOD, per-Oxidase (POD), CAT, and ascorbate per-Oxidase (APX) are important protective enzymes in wheat (Türkan et al., 2005). They can scavenge excess ROS and free radicals generated during cold stress, maintaining the normal growth of wheat (Demiral and Türkan, 2004).

When encountering low temperature cold damage, the loss of water molecules in plant cells increases the concentration of cell fluid, causing damage to the cells themselves. In order to avoid this kind of damage, the body will actively accumulate some soluble substances to increase the cell’s ability to absorb water and maintain normal metabolic functions (Lalk and Dorffling, 1985). The soluble sugar content of wheat is positively correlated with cold resistance, and sucrose and fructose play an important role in the cold resistance of wheat (Kamata and Uemura, 2004; Zeng et al., 2011). The content of proline (Pro) is positively correlated with the cold resistance of wheat. Many results show that when the temperature is low, the proline content in the wheat body rises, more water is aggregated on the protein, preventing the protein from deforming due to dehydration at low temperatures, thereby protecting wheat cells from damage (Bao et al., 2021; Chen et al., 2007).

### 2.2 Molecular mechanism of cold resistance in wheat

**FIGURE 2:**
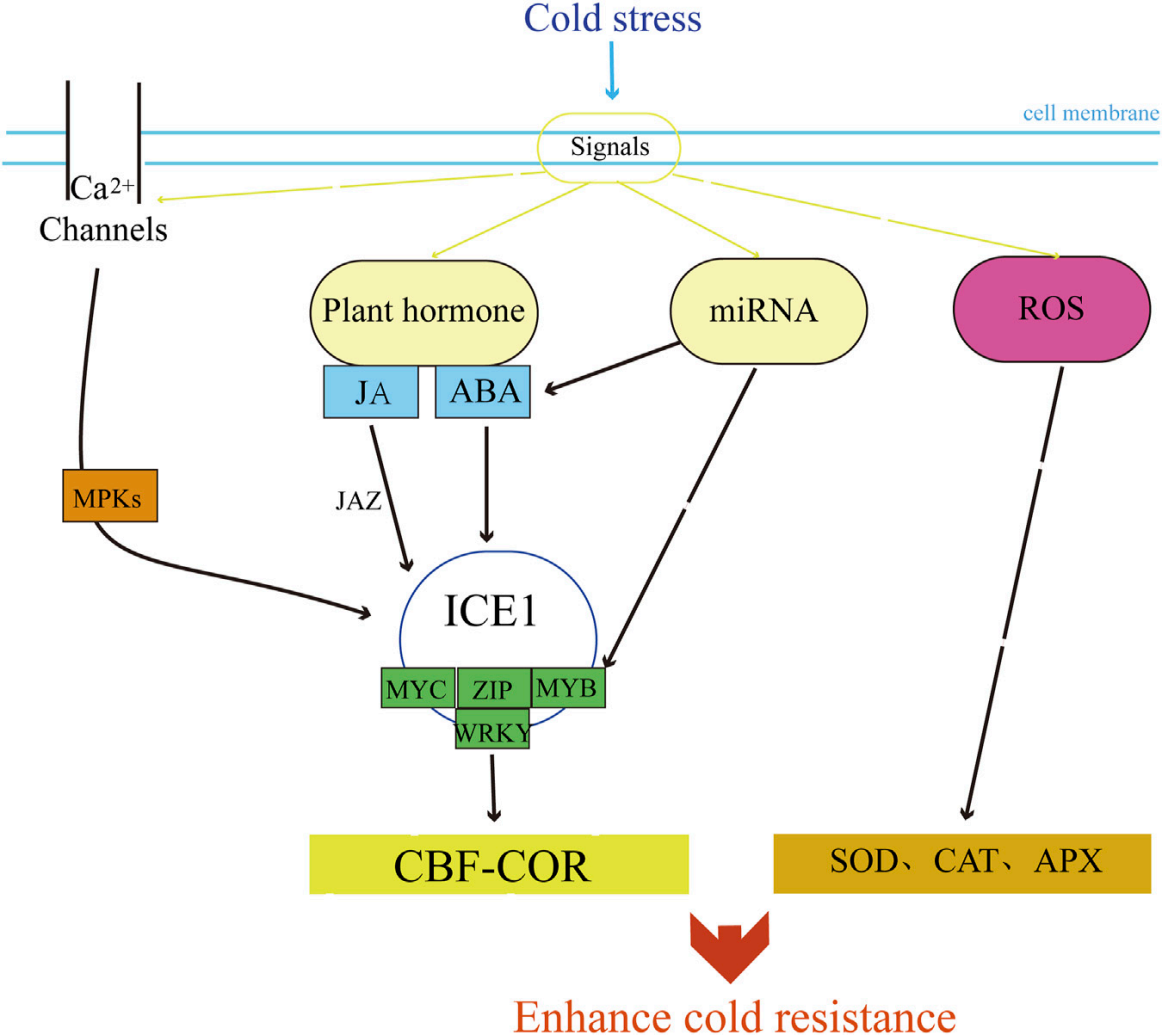
Gene mechanism of ICE-CBF-COR pathway in wheat under cold stress.

Plant cells can respond to low temperature stress by precisely regulating the expression of transcription factors and effector genes. These signaling pathways consist of transcription, translation, and post-transcriptional and post-translational regulatory factors that induce the expression of functional genes in response to cold temperatures. Within one of the signaling pathways is a cis-element known as the C-repeat element/dehydration-responsive element (CRT/DRE), to which C-repeat binding factors (CBF) can bind. This pathway is referred to as the CBF-COR signaling pathway. Studies on a variety of plants have shown that this pathway makes plants have cold resistance by regulating the expression of downstream cold-resistant proteins (Ding et al., 2019). CBF homologous genes in wheat and barley have also been identified (Mohseni et al., 2012; Skinner et al., 2005). *ICE1* (CBF expression inducer) is a MYC-type bHLH transcription factor and is the most characteristic transcription activator of *CBF* genes so far (Chinnusamy et al., 2003; Lee et al., 2005). Overexpression of *ICE1* can increase the expression of *CBFs* (Fursova et al., 2009; Agarwal et al., 2006; Juan et al., 2015). The expression of *ICE1* gene *TaICE41* in wheat is induced by cold stress (Badawi et al., 2008; Guo et al., 2019). The ICE-CBF-COR pathway plays an important regulatory role in the cold resistance pathway of wheat (Gong et al., 2020; Caccialupi et al., 2024) and is considered to be the main cold signal transduction pathway in wheat (Figure 2).

Plant hormones are crucial regulators in perceiving and transmitting various environmental signals and defense responses. Abscisic acid (ABA) and jasmonic acid (JA) are the most studied plant hormones in relation to wheat cold resistance. Lower temperatures can increase endogenous ABA levels in plants, participating in cold stress signal transduction and activating the expression of cold-resistance genes (Zhang et al., 2020; An et al., 2021; Liu et al., 2013). For instance, the cor gene family members such as *cor6.6*, *cor15a*, *cor47*, and *cor78* are highly expressed due to increased ABA levels, thereby enhancing plant freeze tolerance (Hajela et al., 1990). Studies on wheat have found that endogenous ABA content increases during cold acclimation in winter wheat, with a rapid rise in ABA levels inducing the expression of stress-responsive genes and improving freeze resistance (Liu et al., 2013). Another plant hormone, JA, has also been recently discovered to play a regulatory role in plant cold stress responses. Cold stress activates the expression of JA biosynthesis-related genes (AOC, AOS1, AOS2, and LOX2), increasing JA content (Hu et al., 2017; Du et al., 2013). Accumulated JA conjugates with isoleucine to form the active JA-Ile, which is perceived by the COI1-JAZ co-receptor and promotes the ubiquitination and degradation of the repressor protein JAZ *via* the 26S proteasome, thereby activating the ICE-CBF pathway and inducing the expression of certain cold-responsive genes, enhancing plant cold tolerance (Hu et al., 2013).

Excessive ROS production in plant cells under stress conditions is highly reactive and toxic to proteins, lipids, and nucleic acids. ROS in plants primarily exists in the form of free radicals and non-radicals, including hydrogen peroxide (H_2_O_2_), superoxide anion (O^2·-^), hydroxyl radical (OH^·-^), and singlet oxygen (^1^O_2_), *etc.* Elevated ROS levels lead to various harmful cytological effects (Nadarajah, 2020), ultimately resulting in cell damage and death (Gill and Tuteja, 2010). On the other hand, the increase in ROS during stress periods is also considered a signal for the activation of stress response pathways (Baxter et al., 2014). ROS can also function as a signaling molecule, the overaccumulation of ROS at the initial stage of cold stress promotes the activation of antioxidant enzyme genes such as SOD, CAT, and APX to eliminate ROS toxicity. Notably, studies in wheat have demonstrated that the overexpression of the wheat *TaCAT* gene, which eliminates excessive H_2_O_2_, improves the freeze tolerance of transgenic plants (Matsumura et al., 2010).

While calcium (Ca^2+^) is known to enhance stress tolerance (Malko et al., 2023). In plants, low temperatures induce the accumulation of intracellular Ca^2+^ (Yang et al., 2010), and the calcium/calcineurin (Ca^2+^/CaM) regulated receptor kinase CLRK1/2 acts as a sensor for Ca^2+^/CaM under low temperatures. It interacts with and phosphorylates MEKK1, triggering the activation of downstream MPK4/6 and thereby positively regulating the cold response (Furuya et al., 2013). Cold stress activates MKK2, which in turn activates MPK4 and MPK6 (Ichimura et al., 2000). Additionally, cold stress activates protein kinases MPK3 and MPK6, phosphorylates ICE1 protein, and decreases its protein stability and transcriptional activity, thus negatively regulating the expression of CBF and the cold tolerance of plants (Li et al., 2017). Studies in wheat have shown that Ca^2+^ induces cold-responsive genes (*WCOR413, WCOR410, WCOR14*, and *Wrab17*) enhanced cold stress tolerance through maintained cellular redox homeostasis (Malko et al., 2023). Transformation with the Ta MPK6 gene significantly enhances the expression of the ICE-CBF-COR pathway genes in Arabidopsis, improving the plant’s cold tolerance (Yu et al., 2023).

MicroRNA (miRNA) is a class of non-coding small RNA molecules derived from the genomes of eukaryotic organisms, mostly ranging from 21 to 25 nt in length. Their main mode of action is post-transcriptional regulation of target gene expression, which also plays an important role in plant stress response (Naya et al., 2014; Tiwari et al., 2020). Using deep sequencing and bioinformatics prediction methods, miRNAs responding to cold stress have been identified in various crops and model species (Lv et al., 2010; Cao et al., 2014; Sun et al., 2015). In wheat, overexpression of miR399 can positively regulate the protein levels of ICE1 and the expression of key genes *CORs* in the CBF signaling pathway of plants (Peng et al., 2021).

## 3 Application of cold resistant genes and germplasm innovation in wheat breeding

At present, many studies have explained the physiological and molecular mechanisms of cold resistance in wheat from different perspectives, and the research on the genes related to cold resistance in wheat has also progressed rapidly. Cold resistant genes are a class of induced genes that can be activated and expressed in large quantities to produce corresponding cold-resistant substances only at specific periods and conditions. Cold resistance is a complex trait involving many physiological mechanisms, which is the result of the combined action of multiple genes. With the rapid development of molecular biology technology and its wide application in the field of plant breeding, several quantitative trait loci (QTL) related to cold resistance have been mapped by linkage analysis.

Many studies have confirmed that due to the huge genetic background of wheat, wheat cold resistance is controlled and played a role by multiple minor genes. Researchers have mapped allelic loci related to wheat cold resistance on more than 10 pairs of chromosomes such as 1B, 1D, 2B, 2D, 4D, 5A, 5D, and 7A of wheat. The DH population of wheat was used to map QTL for the semi lethal temperature, an index related to cold resistance traits of wheat, and found a total of five major QTL loci controlling the semi lethal temperature, which were distributed on chromosomes 2A, 5A, 1D and 6D of wheat (Båga et al., 2007). Some studies have also found that there are some alleles on chromosomes 5A and 5D that regulate the cold resistance of wheat. At present, QTL mapping loci cover almost all wheat chromosomes, among which 5A and 5D chromosomes are most closely related to cold resistance in wheat (Sutka, 1994; Limin et al.,. 1997), As well as a major-effect QTL related to frost tolerance was reported located on chromosomes 4A recently (Bolouri et al., 2023). The vrnl FRL segment of 5A chromosome in wheat regulates cold resistance in wheat. In common wheat, the major loci controlling cold resistance (*FR-1* and *FR-2*) have been mapped to the long arm of chromosome 5 (Galiba et al., 1995; Tóth et al., 2003). *FR-2* coincides with the *CBFs* gene cluster in wheat and barley (Miller et al., 2006; Francia et al., 2007) and directly induces downstream cor/lea gene expression during cold adaptation (Takumi et al., 2008). A large number of deletions in the CBF cluster of *fr-b2* significantly reduced the cold tolerance of tetraploid and hexaploid wheat (Pearce et al., 2013). A recent study reported that two genes named Wcr-3 and Wcr-4 control cold resistance in wheat, loci on 2B and 2D chromosomes (Lei et al., 2023).

In order to further explore the molecular mechanism of winter wheat response to low temperature stress, several cold resistance genes in wheat were transferred into model plants and proved to affect its cold tolerance. Genes with cold resistance function were cloned from wheat as shown in Table 1. Studies have shown that overexpression of *TabZIP1* reduces the MDA content and electrical conductivity of Arabidopsis at 4°C, and improves the cold tolerance of Arabidopsis (Lv et al., 2018); Overexpression of *TaMYB56-B* transgenic Arabidopsis has a higher survival rate at 4°C, and the expression of *CBF3* and *COR15a* was strongly induced (Zhang et al., 2012). A recent study showed that overexpression of *TaMYB4* enhanced the freezing tolerance of transgenic Arabidopsis, *AtCBF1, AtCBF2, AtCBF3, AtCOR15A, AtCOR47, AtKIN1* and *AtRD29A* in transgenc lines was significantly upregulated (Tian et al., 2023). *TabZIP6* was found as a negative regulator of cold resistance in wheat. The cold tolerance of *Arabidopsis thaliana* overexpressing *TabZIP6* at 4 °C was significantly decreased, and the expression of multiple genes in the CBF-COR cold signaling pathway was downregulated (Cai et al., 2018). Wheat *TaICE41* and *TaICE87* are homologous to Arabidopsis *AtICE1* genes (Badawi et al., 2008). *TaCBF14* and *TaCBF15* play a role in cold tolerance of spring barley, and transgenic *TaCBF14* and *TaCBF15* genes improve the cold tolerance of spring barley (Soltész et al., 2013). Researchers found that *TaMYC2* interacted with *TaICE41*, activated the downstream CBF-COR signaling pathway, and positively regulated the freezing resistance of *Arabidopsis thaliana* (Wang et al., 2022). *TaMPK3* is also involved in regulating the ICE-CBF-COR cold resistance module through its interaction with *TaICE41*, improving freezing tolerance in wheat (Wang et al., 2024). *TaFAD2.8* genes high expression in response to temperature stress (Hajiahmadi et al., 2020). Transgenic *TaMPK6* gene significantly reduced the production and accumulation ofROS in Arabidopsis plants. The expression levels of genes encoding superoxide dismutase (SOD) and catalase (CAT) and the enzyme activities of SOD and CAT were significantly increased. The *CAT* genes were upregulated by cold stress (Ghorbel et al., 2023). Overexpression of *TaCAT* increased the CAT activity of transgenic rice, eliminated excessive accumulation of H_2_O_2_, and improved the cold resistance of transgenic rice (Matsumura et al., 2010). The soluble sugar content of winter wheat seedlings was significantly accumulated (Yu et al., 2008), and the expression of genes encoding key enzymes in glucose metabolism was significantly upregulated. The ectopic expression of wheat *TaFBA-A10* gene was showed in *Arabidopsis thaliana* could increase the content of soluble sugar by affecting the rate of glycolysis and Calvin cycle, thereby improving the cold resistance of *Arabidopsis thaliana* (Zeng et al., 2011); ectopic expression of *TaTPS11*, a gene encoding wheat trehalose-6-phosphate synthase, in Arabidopsis can increase the frost resistance of Arabidopsis by catalyzing the expression level of SnRK1 in sucrose (Peng et al., 2022), and downregulates the expression of this gene with significantly lower cold resistance than Arabidopsis wild-types (Lu et al., 2024). Overexpressing the key enzyme genes *TaG6PDH* and *Ta6PGDH* in the PPP pathway of wheat could improve its cold resistance by scavenging ROS generated at low temperature (Liu et al., 2019; Tian et al., 2021). The mechanism of action of some cold-resistant genes in wheat depends on hormone signal transduction. Lv found that exogenous MeJA treatment can significantly increase the expression of transcription factors (*TabZIP1, TaWABI5, TaMYB80, TaNAC2, TaWRKY80*) in winter wheat under low temperature stress, thereby regulating the CBF signaling pathway to improve cold resistance (Lv et al., 2017; Lv et al., 2018). Chu identified a SA methyltransferase, *TaSAMT1* that converts SA to methyl SA (MeSA) and confers cold tolerance in wheat (Chu et al., 2024). For another example, under low temperature, exogenous ABA increased the expression of *bZIP1, NAC2* and several key enzyme genes of sugar metabolism in wheat (Lv et al., 2018; Liu et al., 2013).

**TABLE 1:** Summary of cold resistance genes in wheat.

Resistance mechanism	Protein types	Cloned genes	References
Increase cell membrane fluidity	FAD (fatty acid desaturase)	*TaFAD2.8,TaFAB2.15*	Hajiahmadi et al. (2020)
Eliminate effects of ROS	CAT catalase	*TaCAT*	Matsumura et al. (2010), Ghorbel et al. (2023)
	Glucose-6-phosphate dehydrogenase	*TaG6PDH*	Liu et al. (2019)
	6-phosphogluconate dehydrogenase	*Ta6PGDH*	Liu et al. (2019)
Increase the content of soluble sugar	FBA (Fructose-1, 6-bisphosphate aldolase)	*Ta FBA-A10*	Zeng et al. (2011)
	TPS (trehalose 6-phosphate synthase)	*TaTPS11*	Peng et al. (2022), Lu et al. (2024)
Transcription factor	MYB	*TaMYB56-B, TaMYB4*	Zhang et al. (2012), Tian et al. (2023)
	MYC	*TaMYC2*	Wang et al. (2022)
	bZIP	*TabZIP1, TabZIP6*	Lv et al. (2018), Cai et al. (2018)
	NAC	*TaNAC2a*,*TANAC4a,TaNAC6*,*TaNAC7*,*TaNAC13*,*TaNTL5*	Lv et al. (2018)
Cold regulated gene	LEA (late embriogenesis abundant protein)	*Wap27,Pvlea218,Wrab17*	Takumi et al. (2008)
	Cor protein	*Wcr3,Wcr4,Wcr719,Wcor80,Wcor14, Wcor72*	Soltész et al. (2013), Lei et al. (2023)
	Wheat cold acclimation protien	*WCS120, WCS19*	Limin et al. (1997)
	ICE	*TaICE41*	Badawi et al. (2008)
Hormone regulation	SAMT (salicylic acid methyltransferase)	*TaSAMT1*	Chu et al. (2024)
Others	MAPK (Mitogen-activated protein kinase)	*TaMPK3, TaMPK6*	Yu et al. (2023), Wang et al. (2024)

## 4 Breeding strategies for cold resistant wheat

**FIGURE 3:**
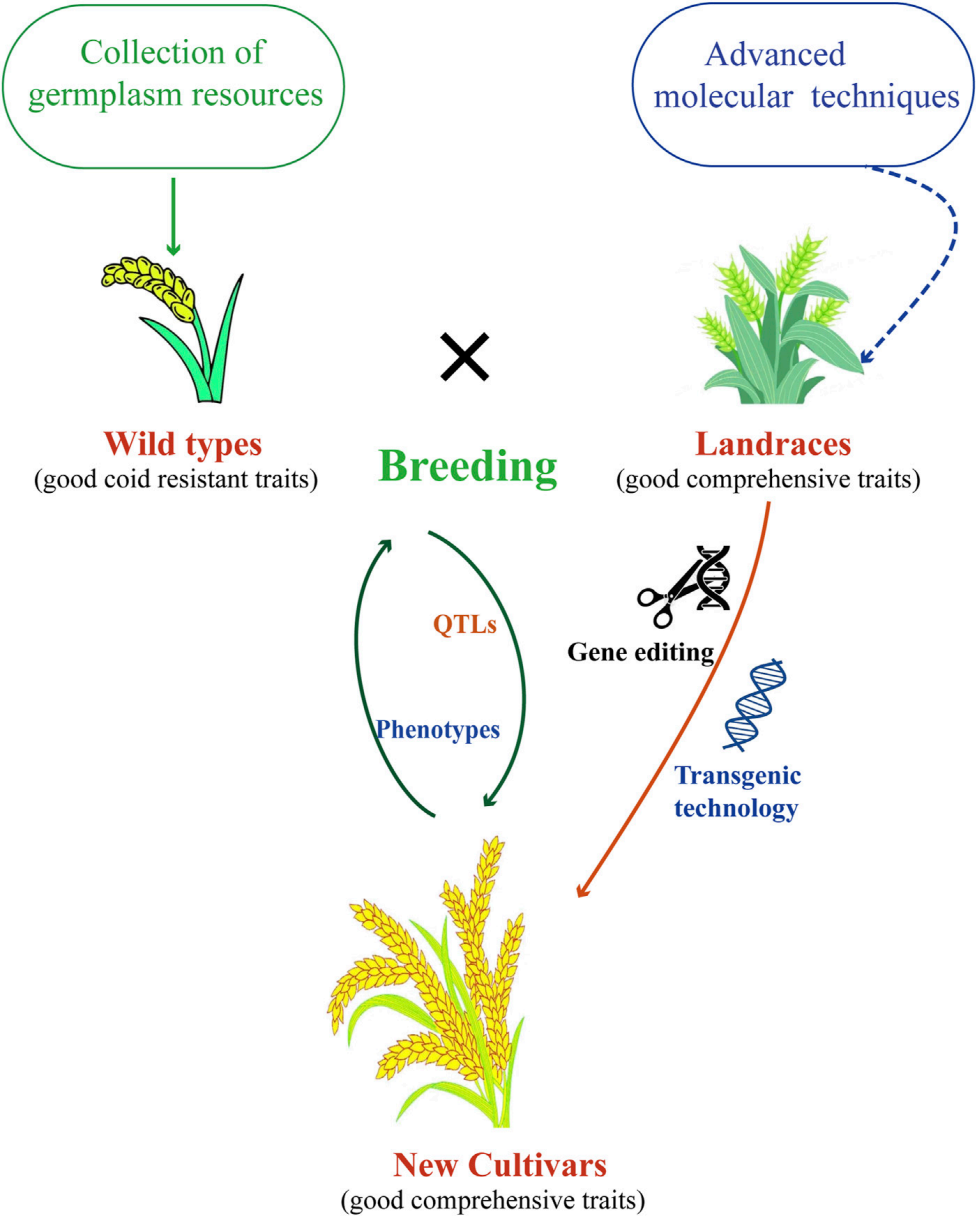
Strategies used for breeding wheat with cold resistance.

Breeding cold-resistant wheat varieties to protect wheat from low-temperature damage and maintain high and stable yields is of significant importance in production. However, breeding for cold resistance in wheat is still at the level of phenotypic selection, the cloned genes were also only functionally characterized in model plants. To make more rational and effective use of cold resistance genes, the following cold resistance breeding strategies for wheat are proposed (Figure 3).

### 4.1 Screening for suitable wheat resources based on cold tolerance traits

Agronomic traits are key factors in wheat cultivars breeding (Tshikunde et al., 2019). Try harder to collect, identify, and study the cold resistance of various germplasm resources (commercial varieties, breeding lines, local varieties, wild relatives, and distant species) is very necessary, and clarify their genetic characteristics and traits of resistance. Only by collecting enough germplasm resources can we explore new diversity of cold resistance genes for cloning, functional verification and aggregation.

There are a number of methods for evaluating and characterising cold resistance criteria in wheat (Valluru et al., 2012; Ou and Wang, 2019). To develop an accurate, fast and scalable method for identifying cold resistance indexes of wheat, so as to judge the cold resistance of breeding materials and germplasm resources through relevant indexes in the field.

### 4.2 QTLs associated with cold resistance

QTLs have great potential for accelerating traditional breeding processes (Jha et al., 2017). Fine mapping and cloning of cold resistance genes/QTLs, studying the mechanism of cold resistance in wheat, and analyzing the regulatory network. Effectively use the existing cold-resistant genes with identification functions, use polymerization hybridization and backcross breeding, combined with molecular marker-assisted selection and generation-adding breeding techniques, accumulate different cold-resistant genes to high-yield varieties with excellent comprehensive traits, and create new materials for high-yield and multi-resistance breeding. On this basis, different hybrid combinations were combined by conventional breeding methods to cultivate new varieties with high yield and durable multi-resistance. Construct various genetic populations and natural populations, and use current wheat genome sequencing and molecular marker chips to carry out the mapping of cold resistance gene QTLs. Develop simple, accurate, and reliable molecular markers and detection technology systems that can be applied in practical breeding.

### 4.3 Functional gene research and new technology applications

In-depth study of key functional genes is a method to improve the yield and quality of crops such as wheat. Genome editing methods, such as CRISPR/Cas9, allowed to manipulate wheat genome to improve agronomic traits (Kim et al., 2018). Transgenic and genome editing was carried out to directly create new varieties of high-yield and high-quality cold-resistant wheat on the basis of the original excellent large varieties. Some technologies in non-biological fields also canbe applied to cold resistance in wheat, such as nanotechnology (Venzhik et al., 2024).
